# Extended-Spectrum *β*-Lactamases among Enterobacteriaceae Isolated from Urinary Tract Infections in Gaza Strip, Palestine

**DOI:** 10.1155/2019/4041801

**Published:** 2019-10-13

**Authors:** Ghassan Tayh, Nahed Al Laham, Houssem Ben Yahia, Rym Ben Sallem, Abed Elkader Elottol, Karim Ben Slama

**Affiliations:** ^1^Laboratoire des Microorganismes et Biomolécules Actives, Faculté des Sciences de Tunis, Université de Tunis El Manar, 2092 Tunis, Tunisia; ^2^Department of Laboratory Medicine, Faculty of Applied Medical Sciences, Al Azhar University-Gaza, Gaza Strip, State of Palestine; ^3^Medical Science Department, University College of Science and Technology-Gaza, Gaza Strip, State of Palestine; ^4^Institut Supérieur des Sciences Biologiques Appliquées de Tunis, Université de Tunis El Manar, 2092 Tunis, Tunisia

## Abstract

**Background:**

Extended-spectrum *β*-lactamase-producing organisms causing urinary tract infections are increasing in incidence and pose a major impendence to health-care facility, having limited therapeutic options. This study aimed to assess the prevalence of ESBLs in Enterobacteriaceae isolates causing urinary tract infections in Gaza strip, Palestine, and to characterize *β*-lactamase types and associated resistance genes.

**Methods:**

Eighty-five Enterobacteriaceae isolates were recovered from urinary tract infections within three months in Gaza Strip hospitals. The characterization of *β*-lactamase genes and the genetic environments of CTX-M, the identification of associated resistance genes, and the presence and characterization of integrons were tested by PCR and sequencing.

**Results:**

The occurrence rate of ESBL among tested isolates was 30 (35.3%), and among ESBL-positive isolates, *bla*_CTX-M_ was the highest followed by *bla*_TEM_. ESBL-CTX-M-1 group was confirmed in 93.3%, and the remaining carried CTX-M-9 group. CTX-M-15, CTX-M-3, CTX-M-1, CTX-M-14, CTX-M-27, and CTX-M-37 enzymes were demonstrated among the isolates with the majority (73%) being CTX-M-15. IS*Ecp-1* was demonstrated in 27 (90%, high incidence) of ESBL isolates. Class 1 integrons have been detected in higher rates (53.3%) in ESBL-positive isolates in comparison with non-ESBL isolates (6, 33.3%). Cassettes of integron-1 contain (*aadA1*, *aadA2*, *aadA5*, *dfrA5*, *dfrA7*, *dfrA12*, and *dfrA17*) genes. The *aac(6′)-Ib-cr* gene was demonstrated in 36.7% of ESBL-positive isolates.

**Conclusions:**

This study indicates that *bla*_CTX-M-15_ was the most prevalent *β*-lactamase in this region. Our study demonstrates for the first time in Palestine the identification of *bla*_CTX-M-15_ in *P. rettgeri* and *S. liquefaciens*, also *bla*_CTX-M-37_ in *E. cloacae.* The coexpression of multiple *β*-lactamase genes with *aac(6′)-Ib-cr* and *qnr* in the presence of IS*Ecp-1* and integrons in individual strains will increase the dissemination of highly resistant strains. ESBL producers were more resistant than non-ESBLs producers for almost all tested antibiotics.

## 1. Introduction

Urinary tract infection (UTI) is one of the most common widespread infections, mainly caused by Enterobacteriaceae, especially *Escherichia coli*, that are encountered by hospitalized and outpatients [1]. Normally, UTIs are treated with different classes of antibiotics such as *β*-lactams, *β*-lactam/*β*-lactamase inhibitors, carbapenems, and fluoroquinolones [2]. However, recent data worldwide reveal that these uropathogens have become resistant to most conventional drugs [3].

Extended-spectrum *β*-lactamases (ESBLs) are bacterial enzymes that hydrolyze oxyimino-cephalosporins and confer resistance to broad-spectrum cephalosporins and aztreonam [4]. Enterobacteriaceae harboring ESBLs is a global problem with limited available treatment options [5]. ESBL-producing bacteria are related with infections that are consequences of bad clinical facilities, inappropriate antibacterial therapy, prolonged hospital stays, and greater hospital costs [6]. In the past years, there has been an increase in widespread dissemination of *β*-lactamase-mediated resistance with high significance in the prevalence of ESBL-producing Enterobacteriaceae [7].

ESBL-producing Enterobacteriaceae have become a major concern in clinical setting worldwide, causing outbreaks related to enzymes of CTX-M class being most common [8]. Recently, ESBLs of CTX-M type have been replaced by SHV- and TEM-ESBLs types worldwide among various members of Enterobacteriaceae [9]. Currently, CTX-M *β*-lactamases are categorized into five classes according to their amino acid sequences and include the CTX-M-1, -2, -8, -9, and -25 clusters [10]. Presently, the dominants of CTX-M-1 and CTX-M-9 are most prevalent in geographical distributions [11–13], with many exhibiting CTX-M-15, which is the most widely distributed CTX-M enzyme worldwide [14–16].

Early investigation of patients infected with ESBL-producing urinary pathogens is necessary to prescribe the most efficient therapy and minimize the dissemination of infection by applying preventive measures. Therefore, the aim of this study was to detect the occurrence of ESBL-producing Enterobacteriaceae and investigate the molecular characteristics of *β*-lactamase-producing isolates obtained from three hospitals in Gaza Strip, Palestine.

## 2. Materials and Methods

### 2.1. Isolation and Identification

Eighty-five Enterobacteriaceae isolates were recovered from urinary tract infection samples obtained in various units of three hospitals in Gaza Strip, Palestine, during the period of three months in 2013. Only one bacterial isolate per patient was included in this study. All collected specimens were sent to the microbiology laboratory to be processed for bacterial isolation and identification. Standard methods for isolation and identification were used [17]. The isolates were confirmed to genus and species level by amplification and sequencing of 16S rRNA gene.

### 2.2. Antibiotic Susceptibility Testing

Susceptibility testing for all isolates to 15 antibiotic agents was performed by the disk diffusion method according to the recommendations of the Clinical and Laboratory Standards Institute (CLSI) [18] using commercial antibiotic disc panels comprising (*μ*g/disk) ampicillin (10), cefoxitin (30), ceftazidime (30), cefotaxime (30), gentamicin (10), amikacin (30), tobramycin (10), amoxicillin-clavulanic acid (20/10), nalidixic acid (30), ciprofloxacin (5), imipenem (10), kanamycin (30), trimethoprim-sulfamethoxazole (1.25/23.75), tetracycline (30), and chloramphenicol (30).

### 2.3. Identification of *β*-Lactamase Genes and Genetic Environment of *bla*_CTX-M_ Genes

PCR amplification and sequencing were used to determine the presence of the genes encoding TEM, SHV, OXA, and CTX-M type *β*-lactamases. The genetic environment surrounding the *bla*_CTX-M_ genes was studied by the amplification of IS*Ecp-1*, *orf477*, and IS*903* [19].

### 2.4. Detection of Non-*β*-Lactam Resistance Genes

Isolates were screened for the genes associated with resistance to tetracycline (*tet(A)* and *tet(B)*), sulfamethoxazole (*sul1*, *sul2*, and *sul3*), and gentamicin (*aac(3)-I*, *aac(3)-II*, and *aac(3)-IV*). The genes *qnrA*, *qnrB*, *qnrS*, *qepA*, *and aac*(6′)-1b were studied by PCR and sequencing to identify the variants according to Jouini et al. [20].

### 2.5. Detection and Characterization of Integrons

The presence of *intI1* and *intI2* genes encoding for classes 1 and 2 integrase, respectively, and the presence of *qacED1*-*sul1* genes within 3′-conserved region of class 1 integrons was identified by PCR. The variable regions of class 1 and 2 integrons were studied in *intI1*- or *intI2*-positive isolates by PCR and sequencing to identify the gene cassettes [19].

Positive and negative controls were kindly provided from the Universite´ Tunis-El Manar, Tunis, and were used in all PCR and sequencing experiments.

### 2.6. Data Analysis

The data of antimicrobial resistance of non-ESBL and ESBLs of Enterobacteriaceae isolates were analyzed by SPSS version 20 software (IBM Corporation, Somers, NY) by applying a Pearson's chi-square test. Level of statistical significance was set at *P* < 0.05.

## 3. Results

### 3.1. Bacterial Strains

In this study, we screened 85 Enterobacteriaceae isolates from urine from three Palestinian hospitals in Gaza Strip for the determination of *β *-lactamase-encoding genes and to characterize their type, genetic environments of CTX-M, and associated resistance genes.

A total of 85 urinary tract infection samples were obtained from Al-Shifa Hospital (37; 43.5%), Balsam Hospital (27; 31.8%), and Al-Remal Center (21; 24.7%). The samples collected from Al-Shifa and Balsam hospitals were from inpatients, and the Al-Remal Polyclinic samples were from outpatients. Out of 85 bacterial isolates, *Escherichia coli* was the predominant isolate (60; 70.6%) followed by *Klebsiella pneumoniae* (15; 17.6%), *Proteus mirabilis* (3; 3.5%), *Enterobacter cloacae* (3; 3.5%), *Serratia liquefaciens* (2; 2.4%), *Providencia rettgeri* (1; 1.2%), and *Morganella morganii* (1; 1.2%).

### 3.2. Antimicrobial Susceptibility and ESBL-Positive Isolates

Among the 85 isolates collected, extended-spectrum *β*-lactamases were confirmed in 30 isolates (35.3%); among these were *E. coli* (20 isolates), *K. pneumoniae* (5 isolates), *E. cloacae* (3 isolates), *S. liquefaciens* (1 isolates) and *P. rettgeri*_._ (1 isolates).

The susceptibility to imipenem was 80.0% against ESBL-producing isolates. However, the resistance rate to cefotaxime and ampicillin was 100%, which was high among ESBL-producing isolates. Resistance to sulfamethoxazole/trimethoprim, nalidixic acid, ciprofloxacin, kanamycin, and tetracycline among strains was 76.7, 66.7, 66.7, 60.0, and 60.0%, respectively. Low resistance in ESBL-producing isolates against gentamicin, chloramphenicol, cefoxitin, amikacin, and amoxicillin clavulanic acid was recorded, and the resistance rate was less than 40.0% (Table 1).

On comparing the antibiotic resistance of ESBL and non-ESBL Enterobacteriaceae, both showed low resistance to imipenem in comparison to other tested antibiotics. However, resistance to other antibiotics was higher among ESBL than non-ESBL isolates with statistical significance (Table 1). To the end, the resistant pattern of ESBL producers was significantly higher than that of non-ESBL producers for all 15 tested antibiotics except for imipenem (Table 1).

### 3.3. Identification of *β*-Lactamases

Molecular characterization of 30 ESBL-producing isolates revealed that all of them harbored *bla*_CTX-M_ genes (100%). *bla*_TEM_, *bla*_OXA_, and *bla*_SHV_ genes were detected in 10 (33.3%), 1 (3.3%), and 1 (3.3%) of the isolates, respectively.

In this study, 28 of the 30 strains were found to be positive for the ESBL-CTX-M-1 group, 23 were confirmed to carry *bla*_CTX-M-15_, 2 strains harbored *bla*_CTX-M-3_, 2 isolates carried *bla*_CTX-M-1_, and the remaining 1 isolate carried *bla*_CTX-M-37_. Of the 2 remaining strains that are positive for the ESBL-CTX-M-9 group, 1 carried *bla*_CTX-M-14_ and 1 harbored *bla*_CTX-M-27_ (Table 2).

*β*-lactamase genes identified by PCR and sequencing among the 20 ESBL-positive *E. coli* strains are as follows: *bla*_CTX-M-15_ (*n* = 11), *bla*_CTX-M-15_, *bla*_TEM-1_ (*n* = 4), *bla*_CTX-M-1_ (*n* = 2), *bla*_CTX-M-14_ (*n* = 1), *bla*_CTX-M-27_ (*n* = 1), and *bla*_CTX-M-3_ (*n* = 1). There were five ESBL-positive *K. pneumoniae* isolates; three isolates harbored (*bla*_CTX-M-15_, *bla*_TEM-1_, *bla*_SHV-1_), one carried (*bla*_CTX-M-15_, *bla*_TEM-1_), and one carried (*bla*_CTX-M-3_). The three isolates of *E. cloacae* producing ESBL had the following *β*-lactamase genes: *bla*_CTX-M-15_, *bla*_TEM-1_, *bla*_OXA-1_ (*n* = 1), *bla*_CTX-M-15_, *bla*_TEM-1_ (*n* = 1), and *bla*_CTX-M-37_ (*n* = 1). The *bla*_CTX-M-15_ was identified in ESBL-producing *Serratia liquefaciens* and *Providencia rettgeri* (Table 2).

Non-ESBL *β*-lactamases were identified in 18 isolates: *E. coli* (*n* = 10), *K. pneumoniae* (*n* = 6), *P. mirabilis* (*n* = 1), and *Morganella* spp. (*n* = 1). The *β*-lactamases that are not classified as ESBL include genes encoding TEM-1, SHV-1, and OXA. *β *-lactamase genes identified among the 18 non-ESBL *β*-lactamase strains are as follows: *bla*_TEM-1_ was the most predominate gene, which was detected in 12 isolates (nine *E. coli*, one *K. pneumoniae*, one *Morganella* spp., and one *P. mirabilis*). The SHV-encoding genes were found among five isolates of *K. pneumoniae* in two variants; *bla*_SHV-1_ and *bla*_SHV-11_ with the frequency of 2 and 3, respectively. Finally, *bla*_OXA-1_ was detected in only one isolate of *E. coli* (Table 2).

### 3.4. Genetic Environments of bla_CTX-M_ Genes

The genetic mechanisms that may be involved in the expression and mobilization of *bla*_CTX-M_ genes and the genetic environments upstream and downstream of *bla*_CTX-M_ genes were studied in ESBL-positive Enterobacteriaceae isolates by PCR and sequencing. The sequence of *orf477* was found downstream of the ESBL-CTX-M-1 group members (*bla*_CTX-M-15_, *bla*_CTX-M-3_, *bla*_CTX-M-1_, and *bla*_CTX-M-27_ genes) in twenty-seven isolates. The insertion sequence (IS*903*) was identified downstream of the *bla*_CTX-M-14_ gene in one isolate (NT134), whereas the downstream region of *bla*_CTX-M-27_ and *bla*_CTX-M-37_ in *E. cloacae* and *E. coli*, respectively, was unknown. The insertion sequence (IS*Ecp-1*) was found upstream *bla*_CTX-M_ genes in twenty-seven isolates; however, the upstream region of *bla*_CTX-M-37_, *bla*_CTX-M-15_, and *bla*_CTX-M-14_ in NT60, NT117, and NT134 isolates, respectively, was unknown.

### 3.5. Integrons and Arrangement of Gene Cassettes

Class 1 integron has been demonstrated in ten ESBL-positive *E. coli* isolates with the following gene cassette arrangements: *dhfr17* + *aadA5* (6 isolates)*, dhfrA7* (2 isolates), and *dfrA12* + *aadA2* (one isolate); three of those integrons lacked the *qacE*Δ*1* and *sul1* genes. In *K. pneumoniae*, class 1 integrons were present in 3 of the 5 ESBL-producing isolates with two different genetic arrangements (*dfrA12* + *aadA2)* and *dfrA5. Int1* was identified in two *ESBL-positive E. cloaca*, the gene cassette implicated in the resistance to streptomycin (*aadA1)* was detected in one isolate, and one of those integrons lacked the *qacE*Δ*1* and *sul1* genes. The cassette that conferred resistance to trimethoprim (*dfrA12*) and streptomycin (*aadA2*) was found in integron-1 among ESBL-producing *S. liquefaciens*.

Five of non-ESBL *E. coli* contained class 1 integron with gene cassette arrangement (*dhfr17* + *aadA5*) found in one isolate. Class 1 integron has been demonstrated in non-ESBL-*Morganella morganii* Isolate (Table 2).

### 3.6. Non-*β*-Lactam Antimicrobial Agent-Coding Genes

A variety of genes which confer resistance to non-*β*-lactam antibiotics were observed among ESBL-producing Enterobacteriaceae: *tetA* and *tetB* (in 11 and 5, respectively, of tetracycline-resistant strains); eleven of the 30 ESBL isolates harbored *sul* genes (*sul1*: (*n* = 7); *sul1* + *sul2*: (*n* = 3); and *sul1* + *sul3*: (*n* = 1)). The *aac(3)-II* gene was found in 13 gentamicin-resistant isolates, and the *aac(6′)-Ib-cr* gene was detected in eleven isolates. The *qnrB1* gene was identified in two isolates, and *qnrA* and *qnrS1* genes were each identified in one isolate. Yet, five non-ESBL *E. coli* isolates harbored some non-*β*-lactam antibiotics coding genes such as *sul-1* (*n* = 2), *aac(3)II* (*n* = 2), *aac(6′)-Ib-cr* (*n* = 1), *tetA* (*n* = 2), *tetB* (*n* = 1), and *sul2* (*n* = 1). Non-ESBL-*Morganella* spp. carried *sul1* gene (Table 2).

## 4. Discussion

A total of 85 Enterobacteriaceae isolates obtained from urinary tract infections from in- and outpatient populations during a three-month time period were evaluated for the production of ESBL, *β*-lactamase enzymes, and associated resistance genes.

The finding showed that among 85 urine isolates, *E. coli* was the most prevalent representing 60 (70.6%) of isolates followed by *K. pneumoniae* 15 (17.6%). This is in agreement with other studies investigating Enterobacteriaceae causing urinary tract infections in Sri Lanka and Qatar [5, 21].

The prevalence of ESBL among our isolates was confirmed in 30 isolates (35.3%). This was similar to the findings of Liu et al., Giwa et al., and Caccamo et al. [22–24]where ESBL production among Enterobacteriaceae causing urinary tract infections was found to be 33.4%, 34.3%, and 36.0%, respectively.

Our findings demonstrated that imipenem was the most effective drug against ESBL isolates. Studies performed in Palestine from different clinical awards demonstrated the same as our findings about the effectiveness of imipenem [1, 25–27].

The results of our study showed that the frequency of antibiotic resistance among ESBL producers is higher than that of resistance in nonproducers; our findings correlate with other studies in Tanzania and India [28, 29]. The spread of ESBL producers in hospitals and their increased antibiotic resistance is alarming.

Resistance to imipenem was found to be low among ESBL producers (20.0%) and non-ESBL producers (12.7%) without statistical significance. The lower resistance to imipenem among ESBL and non-ESBL's was reported in India [30]. The mechanism of resistance to carbapenem occurs by bacterial production of *β*-lactamases and decreases the permeability of the antibiotics by changes in porin channels in the cell wall or reduced susceptibility of bacterial cell toward meropenem through upregulation of efflux pumps [31]. The imipenem resistance in our study could be due to production of carbapenemase. This may be because patients in Palestinian hospitals are treated with carbapenems which may have a role in development of multidrug-resistant strains. Detection of carbapenemase genes are beyond the aims of this study and will be our planned future work.

Molecular genotyping of ESBL-containing isolates revealed the highest presence of CTX-M genes (100%) followed by TEM genes, which is in agreement with previous reports from the region and around the globe [1, 32]. CTX-M enzymes have become dominant extended-spectrum *β*-lactamases in Europe [33] and in many Middle Eastern countries [34].

In this report, 93.3% of Enterobacteriaceae isolates from urine harbored ESBLs-CTX-M-1 group, including *bla*_CTX-M-15_, *bla*_CTX-M-3_, *bla*_CTX-M-1_, and *bla*_CTX-M-37_ genes, whereas 6.7% of the isolates carried the ESBL-CTX-M-9 group, including *bla*_CTX-M-14_ and *bla*_CTX-M-27_. This finding is in agreement with study in Qatar that showed among the ESBL-producing Enterobacteriaceae infections in urine 90% of the CTX-M belonged to CTX-M-1 group and 8% belonged to the CTX-M-9 group [5]. In a recent study in USA, among the ESBL-producing Enterobacteriaceae, 80% and 20% were positive for CTX-M-1 group and CTX-M-9 group, respectively [32].

Genotypic characterization of all ESBL-positive isolates revealed that the majority (73%) of the CTX-M type was CTX-M-15. A lot of reports worldwide described that CTX-M-15 is the most prominent distributed ESBL-CTX-M enzyme [14–16]. Rapid dissemination of CTX-M-15 enzyme is reported in many countries. The easy transfer of this gene is associated with the epidemic plasmid [35].

One of the significant findings of this study is that this is the first report of *bla*_CTX-M-15_ in *P. rettgeri* and *S. liquefaciens* in the Middle East and also the first report of *bla*_CTX-M-15_ in *E. cloacae* in Palestine. In the previous reports, *bla*_CTX-M-15_ was identified in *P. rettgeri* causing urinary tract infections in Croatia [36], and *bla*_CTX-M-15_ was reported *in E*. *cloacae* in Southern China [22], Egypt [11], and Yemen [37].

In our study, CTX-M-15, CTX-M-3, CTX-M-1, CTX-M-14, CTX-M-27, and CTX-M-37 enzymes were demonstrated among the isolates and indicated a diversity of the CTX-M groups in clinical Enterobacteriaceae isolates recovered from Palestinian hospitals. Enterobacteriaceae harboring CTX-M-15 with CTX-M-14 have been reported as the predominant CTX-M-ESBL types in clinical isolates worldwide [11–13]. In a Canadian study that was conducted in 2007, among CTX-M-producing isolates from 11 Canadian medical centers, CTX-M-15 was the most common (86.5%), followed by CTX-M-14, CTX-M-3, and CTX-M-2 [38]. CTX-M-3 was predominant in clinical isolates in Taiwan [39]; this enzyme was described in clinical isolates of Enterobacteriaceae in Korea and France [40, 41]. Sequencing the amplification of PCR products from the two *E. coli* isolates detected the CTX-M-encoding gene as *bla*_CTX-M-1_; this gene has been demonstrated in clinical Enterobacteriaceae in France and Italy [41, 42]. CTX-M-27 enzyme has been reported in nosocomial outbreaks caused by *Salmonella enterica* in a neonatal unit in Tunisia [43].

Our study reported for the first time the presence of CTX-M-37 in clinical Enterobacteriaceae isolates in the Middle East. Its presence was recently reported in clinical strains of *E. cloacae* in Mongolia [44] and was also identified in *S. enterica* serotype Isangi from South Africa [45].

Our results showed that ten *bla*_CTX-M-15_-containing isolates carried more than one *β*-lactamase gene, and three *K. pneumoniae* isolates harbored *bla*_CTX-M-15_, *bla*_SHV-1_, and *bla*_TEM-1_. One isolate coexpressed *bla*_CTX-M-15_ with other *β*-lactamase gene, and *E. cloacae* carried *bla*_CTX-M-15_, *bla*_TEM-1_, and *bla*_OXA-1_. In previous studies, Enterobacteriaceae isolates carrying multiple *β*-lactamase genes have been reported [34, 46, 47]. The occurrence of multiple *β*-lactamase genes in individual strains is a concern because it will enhance coresistance and greater resistance to various classes of antibiotics.

Insertion sequences and integrons have played an important role in the dissemination of *bla*_CTX-M_. IS*Ecp-1* is the most common insertion element associated with *bla*_CTX-M_. Moreover, the role of IS*Ecp-1* is mobilization of the *bla*_CTX-M_ gene, and it acts as a strong promoter which enhances the expression of *bla*_CTX-M_ genes [48]. IS*Ecp-1* was demonstrated in high incidence (90%) among our isolates which is a growing problem of high resistance in our hospitals that can carry high risk through dissemination of *bla*_CTX-M_ between patients. IS*Ecp-1* insertion sequence was found upstream of CTX-M enzyme in Enterobacteriaceae isolates from Tunisia and Croatia [19, 36].

In the present study, class 1 integrons were detected and included resistance genes in the variable region encoding resistance to streptomycin (*aadA1*, *aadA2*, and aadA5) and trimethoprim (*dfrA5*, *dfrA7*, *dfrA12*, and *dfrA17*) which increases the risk of the dissemination of antimicrobial resistance by horizontal transmission by integrons. The integrons lacking *qacE*Δ*1* and *sul1* genes have been previously reported in other countries [19, 49].

ESBL-positive isolates have been demonstrated to carry higher rates (16; 53.3%) of class 1 integrons compared to non-ESBL isolates (6; 33.3%). The integron contribution to transfer extended-spectrum cephalosporin resistance has been established [50]. The frequency of occurrence of integrons among our ESBL-positive isolates was 53% which is less than the rate of isolates from Europe [51]; in contrast to this, the integron frequency was 73% and 92% in isolates from Australia and India [50, 52]. In our study, the percentage of integron in non-ESBL-producing isolates was almost similar to the results obtained from studies performed in Spain and India [52, 53]. May be there are possibilities of transferring the integrons carrying ESBL genes and other drug-resistant determinants from ESBL to non-ESBL-producing isolates that make non-ESBL isolates more resistant and complicate antibiotic resistance problems in our hospitals.

The non-*β*-lactam antibiotic resistance pattern of the isolates showed that the *sul* and *aac(3)-II* genes were the most prominent identified in our isolates. This is in agreement with the findings of a study conducted in Spain [54]. The variant *aac(6′)-Ib-cr* gene has the ability to reduce susceptibility to aminoglycosides and ciprofloxacin [16]. This gene prevalence rate in our study was 36% among ESBL-producing Enterobacteriaceae isolates in comparison with 25% in a previous study in Palestine [55]. A significant association between resistance to broad-spectrum cephalosporins and resistance to quinolones was reported [56]. The association of *qnrs1* gene with *bla*_CTX-M_ identified in this study was similar to the findings of a study in Algeria [57]. We also observed the associations between *qnrB1* and *bla*_CTX-M_ which is in agreement with previously reported results in Tunisia [58]. Moreover, the correlation between *qnrA* and *bla*_CTX-M_ demonstrated here was also reported in Yemen [37]. The presence of *β*-lactamases in association with other resistance genes, *qnr* gene and *aac(6′)-Ib-cr*, in the same strain could complicate the treatment of these pathogens. These results support previous reports that suggest larger dissemination of *aac(6′)-Ib-cr* with *qnr* determinants, in ESBL-producing isolates harboring *bla*_CTX-M-15_ gene [58–60].

Regarding the comparison of the occurrence of non-*β*-lactam genes within ESBL and non-ESBL isolates, the non-*β*-lactam genes were confirmed in 83% and 33.3% in ESBL and non-ESBL isolates, respectively. These findings could complicate the resistance problem and limit the antimicrobial drug therapy of infections caused by ESBL producers.

## 5. Conclusions

Our study highlights the high incidence of ESBL in Enterobacteriaceae recovered from urinary tract infections in Gaza hospitals, Palestine, with the increase in antibiotics resistance for most commonly used antibiotics in our hospitals except for imipenem that showed the highest activity against ESBL isolates. ESBL producers were found to be more resistant than non-ESBLs producers for almost all tested antibiotics. This study indicates that *bla*_CTX-M-15_ was the most prevalent *β*-lactamase in Gaza Strip hospitals. To our knowledge, this is the first report on identification of *bla*_CTX-M-15_ in *P. rettgeri* and *S. liquefaciens* and also *bla*_*CTX-M-37*_ in *E. cloacae* in the Middle East.

The associations between multiple *β*-lactamase genes in individual strains with other resistance genes such as *aac(6′)-Ib-cr* and *qnr* in the presence of IS*Ecp-1* and integrons increase the dissemination of highly resistant strains and complicate the problem of clinical conditions and treatment failure in our hospitals.

## Figures and Tables

**Table 1:** Comparison of antibiotics resistance in 30 ESBL and 55 non-ESBL Enterobacteriaceae.

Antibiotics	Resistance in ESBL-producing isolates (%)	Resistance in non-ESBLs-producing isolates (%)	*P* value
Cefotaxime	30 (100%)	6 (11%)	0.000
Ceftazidime	15 (50%)	4 (7.3%)	0.000
Gentamicin	10 (33.3%)	3 (5.5%)	0.001
Ampicillin	30 (100%)	32 (58.2%)	0.000
Imipenem	6 (20.0%)	7 (12.7%)	0.278
Nalidixic acid	20 (66.7%)	7 (12.7%)	0.000
Sulfamethoxazole/trimethoprim	23 (76.7%)	18 (32.7%)	0.000
Tobramycin	15 (50.0%)	5 (9.1%)	0.000
Ciprofloxacin	20 (66.7%)	5 (9.1%)	0.000
Kanamycin	18 (60.0%)	9 (16.4%)	0.000
Tetracycline	18 (60.0%)	2 (3.6%)	0.000
Cefoxitin	10 (33.3%)	1 (1.8%)	0.000
Amikacin	10 (33.3%)	3 (5.5%)	0.001
Amoxicillin-clavulanic acid	10 (33.3%)	3 (5.5%)	0.001
Chloramphenicol	12 (40.0%)	7 (12.7%)	0.012

**Table 2:** Characteristics of the 30 ESBL and 18 non-ESBL Enterobacteriaceae isolates recovered from urinary tract infections.

Class 1 integron			Non-*β*-lactamase genes	Genetic environment of *bla*_CTX-M_ gene		*β*-lactamase genes	Antimicrobial resistance pattern	Hospital	Species	Bacterial code
Variable region	*qacE*∆1 + *sul1*	*Int1*		Upstream region	Downstream region					
*ESBLs*										
−	−	−	*aac(3)II, aac(6′)-Ib-cr, sul2, QnrS1*	IS*Ecp-1*	*orf477*	*bla*_CTX-M-15_, *bla*_TEM-1_	CTX, GM, AMP, IMP, NAL, SXT, TOB, CIP	Al-Shifa	*K. pneumoniae*	NT13
*dhfr17* + *aadA5*	+	+	*sul1*	IS*Ecp-1*	*orf477*	*bla*_CTX-M-15_	CTX, AMP, SXT, CIP	Al-Shifa	*E. coli*	NT14
−	−	−	*aac(3)II, aac(6′)-Ib-cr, sul1, TetB*	IS*Ecp*	*orf477*	*bla*_CTX-M-15_	CAZ, CTX, GM, AMP, KAN, NAL, SXT, TOB, CIP, TET	Balsam	*E. coli*	NT40
−	−	−	*qnrA, sul1*	IS*Ecp-1*	*orf477*	*bla*_CTX-M-15_	CTX, AMP, NAL, SXT, CIP	Al-Shifa	*E. coli*	NT55
*dfrA12* + *aadA2*	+	+	*aac(6′)-Ib-cr, sul1*	IS*Ecp-1*	*orf477*	*bla*_CTX-M-15_, *bla*_TEM-1_, *bla*_SHV-1_	CAZ, CTX, AMP, KAN, NAL, AMK, SXT, TOB, CIP	Al-Shifa	*K. pneumoniae*	NT57
−	−	−	*sul1, TetA*	IS*Ecp-1*	*orf477*	*bla*_CTX-M-15_	CAZ, CTX, AMP, IMP, NAL, SXT, CIP, TET	Al-Shifa	*E. coli*	NT58
−	−	+	*aac(3)II, qnrB1, TetA*	Unknown	Unknown	*bla*_CTX-M-37_	AMC, CAZ, CTX, GM, AMP, IMP, KAN, NAL, AMK, SXT, FOX, TOB, CIP, TET	Al-Shifa	*E. cloacae*	NT60
−	−	−		IS*Ecp-1*	*orf477*	*bla*_CTX-M-15_, *bla*_SHV-1_, *bla*_TEM-1_	AMC, CAZ, CTX, GM, AMP, KAN, NAL, AMK, SXT, TOB, CIP, TET	Al-Shifa	*K. pneumoniae*	NT66
*dfrA12* + *aadA2*	+	+	*aac(3)II, aac(6′)-Ib-cr, qnrB1, sul1, sul2, TetA*	IS*Ecp-1*	*orf477*	*bla*_CTX-M-15_	CAZ, CTX, AMP, NAL, CHL, CIP, TET	Al-Shifa	*E. coli*	NT68
−	−	−		IS*Ecp-1*	*orf477*	*bla*_CTX-M-15_, *bla*_TEM-1_	AMC, CTX, AMP, FOX	Al-Shifa	*Enterobacter cloacae*	NT69
*dhfr17* + *aadA5*	−	+	*aac(3)II, aac(6′)-Ib-cr, sul1, sul3, TetA, TetB*	IS*Ecp-1*	*orf477*	*bla*_CTX-M-15_	AMC, CAZ, CTX, GM, AMP, KAN, NAL, SXT, FOX, TOB, CHL, CIP, TET	Al-Shifa	*E. coli*	NT71
*dfrA12* + *aadA2*	+	+	*aac(3)II, aac(6′)-Ib-cr, sul1, TetA*	IS*Ecp-1*	*orf477*	*bla*_CTX-M-15_	AMC, CAZ, CTX, GM, AMP, KAN, NAL, AMK, SXT, FOX, TOB, CHL, CIP, TET	Al-Shifa	*Serratia liquefaciens*	NT73
−	−	−		IS*Ecp-1*	*orf477*	*bla*_CTX-M-15_	CTX, AMP, KAN, AMK, FOX, TOB	Al-Shifa	*E. coli*	NT75
−	−	−	*aac(3)II, sul1, sul2, TetA*	IS*Ecp-1*	*orf477*	*bla*_CTX-M-15_, *bla*_TEM-1_	CTX, GM, AMP, SXT, TOB, TET	Al-Shifa	*E. coli*	NT84
*dhfr17* + *aadA5*	+	+	*aac(3)II, aac(6′)-Ib-cr, sul1, sul2*	IS*Ecp-1*	*orf477*	*bla*_CTX-M-3_	AMC, CAZ, CTX, AMP, KAN, NAL, SXT, FOX, TOB, CHL, CIP	Al-Shifa	*E. coli*	NT92
*dhfr17* + *aadA5*	−	+	*aac(3)II, aac(6′)-Ib-cr, sul1, TetB*	IS*Ecp-1*	*orf477*	*bla*_CTX-M-15_	AMC, CAZ, CTX, GM, AMP, KAN, NAL, SXT, FOX, TOB, CHL, CIP, TET	Al-Shifa	*E. coli*	NT98
−	−	+	*aac(6′)-Ib-cr, qnrS1, TetB*	IS*Ecp-1*	*orf477*	*bla*_CTX-M-15_, *bla*_SHV-1_, *bla*_TEM-1_	CAZ, CTX, AMP, IMP, KAN, SXT, CHL, CIP, TET	Balsam	*K. pneumoniae*	NT106
−	−	−	*TetA*	IS*Ecp-1*	*orf477*	*bla*_CTX-M-15_	AMC, CAZ, CTX, AMP, NAL, SXT, FOX, TET	Al-Remal	*E. coli*	NT108
*dfrA5*	−	+	*aac(3)II*	IS*Ecp-1*	*orf477*	*bla*_CTX-M-3_	AMC, CAZ, CTX, AMP, IMP, KAN, AMK, SXT, TOB, CHL	Al-Remal	*K. pneumoniae*	NT112
−	−	−	*TetB*	IS*Ecp-1*	*orf477*	*bla*_CTX-M-15_, *bla*_TEM-1_	CAZ, CTX, AMP, SXT, TET	Al-Remal	*E. coli*	NT116
*dfrA7*	−	+	*TetA*	Unknown	*orf477*	*bla*_CTX-M-15_, *bla*_TEM-1_	AMC, CTX, AMP, NAL, SXT, FOX, TET	Balsam	*E. coli*	NT117
*dhfr17* + *aadA5*	−	+	*aac(3)II, aac(6′)-Ib-cr, sul1, TetA*	IS*Ecp-1*	*orf477*	*bla*_CTX-M-15_	CAZ, CTX, AMP, KAN, NAL, AMK, SXT, TOB, CIP, TET	Balsam	*E. coli*	NT121
−	−	−	*TetA*	IS*Ecp-1*	*orf477*	*bla*_CTX-M-1_	CTX, AMP, KAN, NAL, CHL, CIP, TET	Balsam	*E. coli*	NT126
*dhfr17* + *aadA5*	−	+		IS*Ecp-1*	*orf477*	*bla*_CTX-M-15_	CTX, AMP, AMK, SXT	Balsam	*E. coli*	NT129
−	+	+	*aac(3)II, aac(6′)-Ib-cr, sul1*	Unknown	*IS903*	*bla*_CTX-M-14_	CTX, GM, AMP, KAN, NAL, AMK, SXT, TOB, CHL, CIP, TET	Balsam	*E. coli*	NT134
*dhfrA7*	+	+	*sul1*	IS*Ecp-1*	*orf477*	*bla*_CTX-M-15_, *bla*_TEM-1_	CTX, AMP, KAN, NAL, SXT, CIP	Balsam	*E. coli*	NT138
−	−	−		IS*Ecp-1*	*orf477*	*bla*_CTX-M-15_	CTX, AMP, CHL	Balsam	*Providencia rettgeri*	NT146
−	−	−	*TetA*	IS*Ecp-1*	Unknown	*bla*_CTX-M-27_	CTX, AMP, KAN, NAL, AMK, CIP, TET	Balsam	*E. coli*	NT147
−	−	−	*TetA*	IS*Ecp-1*	*orf477*	*bla*_CTX-M-1_	CTX, AMP, KAN, NAL, CHL, CIP, TET	Balsam	*E. coli*	NT151
*aadA1*	+	+	*aac(3)II, sul1, sul2*	IS*Ecp-1*	*orf477*	*bla*_CTX-M-15_, *bla*_TEM-1_, *bla*_OXA-1_	CTX, GM, AMP, IMP, KAN, SXT, FOX, TOB, CHL	Al-Shifa	*Enterobacter cloacae*	NT157

*Non-ESBLs*										
−	−	−				*bla*_TEM-1_	CAZ, CTX, AMP, CHL	Al-Shifa	*E. coli*	NT4
−	+	+	*aac(3)II, sul1*			*bla*_TEM-1_	GM, AMP, KAN, NAL, SXT, TOB, CIP	Al-Shifa	*E. coli*	NT16
						*bla*_SHV-11_	AMP	Al-Remal	*K. pneumoniae*	NT25
−	−	+	*sul1, sul2*			*bla*_TEM-1_	AMP, NAL, SXT, CIP	Al-Remal	*E. coli*	NT28
*dhfr17* + *aadA5*	+	+	*sul1*			*bla*_TEM-1_	AMP, SXT	Al-Remal	*E. coli*	NT29
−	−	−				*bla*_TEM-1_	AMP	Al-Remal	*E. coli*	NT30
−	−	−				*bla*_SHV-11_	AMP	Balsam	*K. pneumoniae*	NT36
−	−	−	*aac(3)II, aac(6′)-Ib-cr, TetA, TetB*			*bla*_OXA-1_	GM, AMP, KAN, NAL, TOB, CIP, TET	Al-Shifa	*E. coli*	NT64
−	+	+	*sul1*			*bla*_TEM-1_	AMC, AMP, IMP, SXT	Al-Shifa	*Morganella morganii*	NT89
−	−	−				*bla*_SHV-11_	AMP	Al-Remal	*K. pneumoniae*	NT102
−	−	+				*bla*_TEM-1_	AMC, AMP, KAN, SXT, CHL	Al-Remal	*E. coli*	NT107
−	−	−				*bla*_SHV-1_	AMP	Al-Remal	*K. pneumoniae*	NT114
−	−	+				*bla*_TEM-1_	AMP, NAL, SXT	Al-Remal	*E. coli*	NT115
−	−	−				*bla*_TEM-1_	CTX, AMP, SXT	Balsam	*E. coli*	NT123
−	−	−				*bla*_TEM-1_	AMP, IMP, KAN, CHL	Balsam	*Proteus mirabilis*	NT125
−	−	−				*bla*_TEM-1_	AMP	Balsam	*E. coli*	NT130
−	−	−				*bla*_TEM-1_	CAZ, CTX, AMP, IMP	Balsam	*K. pneumoniae*	NT131
−	−	−				*bla*_SHV-1_	CAZ, CTX, AMP, IMP	Balsam	*K. pneumoniae*	NT149

## Data Availability

All data used to support the findings of the study are approved and included within the article.
